# Identification of SNPs Associated with Drought Resistance in Hybrid Populations of *Picea abies* (L.) H. Karst.–*P. obovata* (Ledeb.)

**DOI:** 10.3390/genes15111440

**Published:** 2024-11-07

**Authors:** Yulia Vasileva, Andrei Zhulanov, Nikita Chertov, Yana Sboeva, Svetlana Boronnikova, Victoria Pechenkina, Yulia Nechaeva, Ruslan Kalendar

**Affiliations:** 1 Faculty of Biology, Perm State University, 614990 Perm, Russia; yulianechaeva@mail.ru (Y.V.); aumakua.ru@gmail.com (A.Z.); syper.gall@mail.ru (N.C.); yana_prishnivskaya@mail.ru (Y.S.); p_viktoria2@mail.ru (V.P.); ulia-2012@mail.ru (Y.N.); 2 Perm Agricultural Research Institute, Branch of Perm Federal Research Center Ural Brunch Russian Academy of Sciences, 614990 Perm, Russia; 3 National Laboratory Astana, Nazarbayev University, Astana 010000, Kazakhstan; 4 Institute of Biotechnology HiLIFE, University of Helsinki, 00014 Helsinki, Finland

**Keywords:** adaptive-significant genes, SNP, nucleotide diversity, drought resistance, *P. abies*–*P. obovata* spruce complex, the Urals

## Abstract

Background/Objectives: The spruces of the *Picea abies*–*P. obovata* complex have a total range that is the most extensive in the world flora of woody conifers. Hybridization between the nominative species has led to the formation of a wide introgression zone, which probably increases the adaptive potential of the entire species complex. This study aimed to search the genes associated with drought resistance, develop primers for the informative loci of these genes, identify and analyze SNPs, and establish the parameters of nucleotide diversity in the studied populations. Methods: The objects of this study were eight natural populations of the spruce complex in the Urals. Nucleotide sequences related to drought resistance spruce genes with pronounced single-nucleotide substitutions were selected, based on which 16 pairs of primers to their loci were developed and tested. Results: Based on the developed primers, six pairs of primers were chosen to identify SNPs and assess the nucleotide diversity of the studied populations. All selected loci were highly polymorphic (6 to 27 SNPs per locus). It was found that the *Pic01* locus is the most variable (*Hd* = 0.947; *π* = 0.011) and selectively neutral, and the *Pic06* locus is the most conservative (*Hd* = 0.516; *π* = 0.002) and has the most significant adaptive value. Conclusions: The nucleotide diversity data for the studied populations reveal similar values among the populations and are consistent with the literature data. The discovered SNPs can be used to identify adaptive genetic changes in spruce populations, which is essential for predicting the effects of climate change.

## 1. Introduction

Modern climate changes, primarily associated with rising temperatures and fluctuations in precipitation, make the issue of the impact of these changes on forests relevant and necessitate the development of a strategy for preventive adaptation measures in forest management [1,2]. Forest plantations perform a climate-regulating function both locally and globally. At the same time, they are objects exposed to climate impacts. For species with a continuous range, the effects of climate change will be differentiated, being associated with the geographical location of the population and the climatic characteristics under which they were formed [3]. Intra- and interspecific differentiation will play a significant role in adapting forest species to climate change [4]. In the context of global climate change, forest ecosystems are primarily affected by elevated temperatures, which, especially in combination with drought, affect all plant life processes. In response to water stress, numerous signaling pathways and response mechanisms are activated to counteract water loss and adapt to emerging threats [5]. Changes in metabolism associated with rising air temperatures have been observed in various tree species. For example, the vital condition of the European spruce, which grows in Eastern Europe, has significantly worsened in recent years. The possible cause is primarily increased spring and summer temperatures and decreased precipitation [6,7].

Some of the most widespread woody conifers are species of the spruce genus (*Picea*), the total range of which is one of the most extensive in the world flora of woody conifers [8]. Spruce trees common in Northern Eurasia are represented mainly by two species—*Picea abies* (L.) Karst. and *Picea obovata* (Ledeb.)—which form natural (introgressive) hybrids of *P. abies*–*P. obovate* [9]. Thus, earlier, E.G. Bobrov put forward a theory of introgressive hybridization of Eastern European spruce trees, according to which, as a result of the processes of introgressive hybridization of European and Siberian spruce trees, a large complex of populations of hybrid origin was formed, and it located, among other things, in the Urals: the region studied [10,11]. The hierarchical system of populations of these spruce species has become an example for studying the microevolution of interconnected gene pools of closely related species under dynamic environmental transformations. These changes occurred in the Pleistocene and early Holocene when the most drastic transformations of spruce habitats occurred [12,13,14,15]. The modern distribution of Norway spruce and Siberian spruce has a clear direction from west to east. However, it is impossible to draw an exact boundary between their natural ranges; they occupy different, albeit overlapping, ecological niches [16]. Dynamics are still observed today due to the adaptation of spruce species and hybrid complexes to global climate shifts [17]. At the same time, hybridization between the nominative species probably increases the adaptive potential of the entire species complex due to the interaction of the gene pools of the parental species [18]. At the junction of the evolutionary migration paths of spruces in the postglacial period, modification (adaptive) genotypes could have appeared. Their reaction to climate change is of interest from the point of view of studying the possibility of adaptation of these genotypes to climate change and forecasting probable migration directions in the long term.

Challenges to conifer populations associated with global climatic and anthropogenic changes in the environment draw attention to the identification of adaptive genetic variability based on modern genomic data using approaches such as the analysis of outlier loci (ecological or by the level of genetic differentiation) among the single-nucleotide polymorphisms (SNPs) localized in genes with a known function in specific biochemical and physiological processes. Testing SNPs associated with key fitness traits and various environmental factors is a promising direction for identifying the genetic basis of adaptation in spruces. The study of this aspect is the most interesting in the Urals, in the zone of active introgression of two spruce species.

This study aimed to search for regions of adaptively significant genes that are associated with drought resistance and potentially highly polymorphic, develop primers for the most informative loci of these genes, and identify and analyze informative SNP markers to establish the parameters of nucleotide diversity in populations of the *P. abies–P. obovata* spruce complex in the Urals (Perm region).

## 2. Materials and Methods

### 2.1. Sample Collection and DNA Extraction

**Figure 1:**
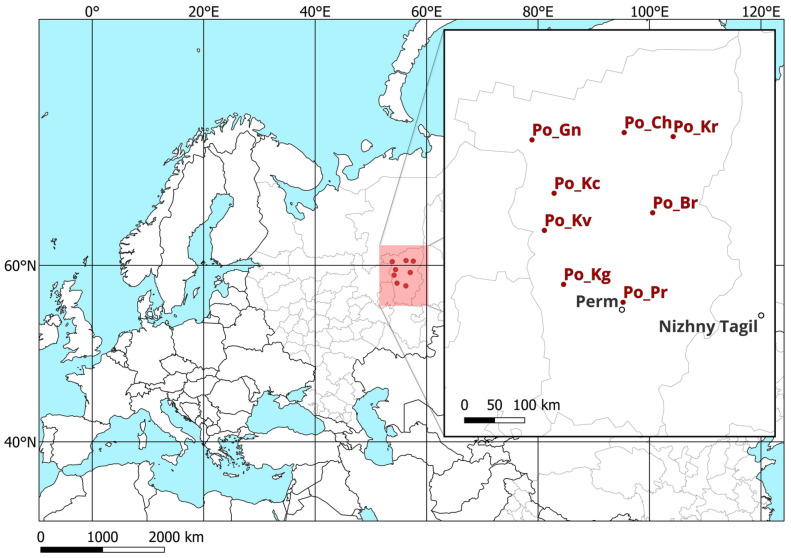
Location diagram of the studied populations of the *P. abies–P. obovata* complex. *Po_Ch*—Cherdynsky district, *Po_Kr*—Krasnovishersky district, *Po_Gn*—Gainsky district, *Po_Kc*—Kochevsky district, *Po_Br*—Usolsky district, *Po_Kv*—Kudymkarsky district, *Po_Kg*—Karagaysky district, and *Po_Pr*—Permsky district of the Perm region.

The material for the study was needle samples collected from 8 natural populations of the *P. abies*–*P. obovata* spruce complex in the Perm region, Russia (from 53°9′ to 57°7′ N and from 58°0′ to 60°5′ E) within the Middle and Northern Urals. The minimum distance between populations was 58 km, and the maximum was 283 km (Figure 1; Supplementary Table S1).

The needles were collected individually from 30 to 31 trees in each of the eight populations (246 trees in total) located at least 150 m from each other. Then, 8 random samples (64 samples) were selected from each population to analyze the polymorphisms of adaptively significant genes. DNA was isolated from the dried material using an acidic CTAB solution with further purification using a high-salt gel electroelution trap [19,20].

### 2.2. Amplification and Sequencing

For PCR amplification, a 10 μL reaction mixture was used, containing 1 μL of 10× PCR buffer; 2.5 mM MgCl2; 0.2 mM of each dNTP (Evrogen, Moscow, Russia); 0.2 μM each for forward and reverse primers; 0.5 units of Taq polymerase (Syntol, Moscow, Russia); and 10 ng of total DNA. Amplification was carried out in a C1000 Thermal Cycler (Bio-Rad, Hercules, CA, USA) according to the following program: 5 min at 94 °C, the subsequent 30 cycles at 94 °C for 30 s, Ta °C for 45 s, and 72 °C for 2 min, and 72 °C for 10 min. To control the amplification of a single fragment, the amplification products were separated using electrophoresis in a 2% agarose gel and visualized in the GelDoc XR gel documentation system (Bio-Rad, Hercules, CA, USA). Enzymatic purification of PCR products for further sequencing was performed using a mixture of Exo I and FAST-AP enzymes (Thermo Fisher Scientific Inc., Waltham, MA, USA). The BigDye^®^ Terminatorv3.1 Cycle Sequencing Kit (Applied Biosystems, Waltham, MA, USA) was used for the sequencing reaction; the direct sequence of the PCR primer pair was used as a primer. The sequencing reaction products were purified from excess fluorescently labeled nucleotides using the BigDye^®^ X Terminator Purification Kit (Applied Biosystems, Waltham, MA, USA). Capillary electrophoresis of the sequencing reaction products was performed at Perm State University (Perm, Russia) on a Genetic Analyzer 3500xl using the POP7 matrix (Applied Biosystems, Waltham, MA, USA). The nucleotide sequences of each selected locus were sequenced for eight trees from each study population.

Preliminary processing of the obtained data was performed with the Sequence Scanner v2.0 program (Applied Biosystems, Waltham, MA, USA), and multiple alignment of nucleotide sequences was performed with the Unipro U-GENE v1.50 program [21] using the MAFFT algorithm [22]. The sequenced loci were compared with those available in the NCBI genetic database using BLAST 2.2.26+ (https://blast.ncbi.nlm.nih.gov (accessed on 1 August 2024)) [23] and UniProtKB tools (https://www.uniprot.org/blast (accessed on 1 August 2024)).

### 2.3. Locus Selection and Primer Development for Highly Polymorphic Regions of Adaptively Significant Genes

The search for genes or genetic determinants associated with drought resistance was carried out in genetic databases (NCBI, Ensembl Plants, Plant Constituents, Tree Genes, UniProt KB, Plaza, BioProject, Gene Ontology, DDBJ, KEGG, European Genome-phenome Archive, Sequence Read Archive, etc.). For each of the selected gene families, the corresponding identifiers were found in the Gene Ontology database (GO, https://geneontology.org/ (accessed on 1 August 2024)). A bioinformatics search for nucleotide sequences of the genes of interest was performed (description can be found in the Section 3). A set of polymorphic regions in the nucleotide sequences of adaptively significant genes of *Picea* species belonging to one or more GO terms and associated with drought resistance was selected based on the genome-wide data of single-nucleotide polymorphisms (SNPs) found for *Picea abies* [24]. The primers were developed using FastPCR software v.6.9 [25] with the following parameters: the SNP-containing fragment to be amplified was 400–600 nucleotides, the optimal primer length was 20 nucleotides, and the optimal annealing temperature was 60 °C. The primer pairs that did not form dimers or non-specific amplification products were selected. In silico PCR analysis for primer pairs for the Picea genomes was performed using virtualPCR v.1.0 [26]. A search was performed in the *Picea abies* genome [27] taken from the Tree Genes database (DB, https://treegenesdb.org/FTP/Genomes/Paab/v1.0b/genome/Paab.1_0b.fa.gz (accessed on 1 August 2024)) to verify the uniqueness of the sequences amplified by the primers. After their selection, the most specific primers were amplified from spruce DNA (Table 1).

### 2.4. Determination of Polymorphism of Adaptively Significant Genes

Haplotypes were reconstructed in the DnaSP program [28]. The following nucleotide polymorphism indices were calculated based on a comparison of their nucleotide sequences: the number of variable sites (*S*); the number of haplotypes in the population (*h_n_*); the total haplotype diversity (*Hd*) according to Nei [29]; the nucleotide diversity (*π*), which estimates the average number of pairwise differences between two sequences per site; the nucleotide diversity parameter (per site), calculated based on the number of mutations (*θ_W_*); or the Watterson estimate [30]. To assess the compliance of the nature of nucleotide substitutions with the neutrality hypothesis, the Tajima D-test [31] was performed for each locus (*D_T_*), i.e., the degree of neutrality of the existing polymorphism concerning natural selection was checked.

## 3. Results

### 3.1. Locus Selection and the Development of the Primer for Highly Polymorphic Regions of Adaptively Significant Genes

Currently, the global genetic databases have accumulated a lot of data related to genes or genetic determinants associated with drought resistance: NCBI contains 265 annotated genes, 25,082 nucleotide sequences, 10,030 amino acid sequences, and over 79,000 scientific publications (https://www.ncbi.nlm.nih.gov/search/all/?term=drought+tolerance (accessed on 1 August 2024)). As of December 2023, there were 166 records for *Picea abies* in the NCBI Gene DB, while the gene sequences of *P. obovata* were absent in that DB. Based on previous studies [32], the prominent gene families associated with drought resistance in coniferous woody plants were selected, namely dehydrin family genes (DHN); late embryogenesis protein group genes (LEA); heat-shock protein class genes (HSP); genes of osmoprotective carbohydrate synthesis and transport proteins; proline synthesis genes; genes of oxidative stress protection proteins; and genes of ethylene and abscisic acid metabolic pathways. Too broad categories, such as transcription factors, ribosomal genes, photosynthesis genes, etc., were excluded from consideration. The corresponding GO terms were found for each of the selected gene families. The identifiers listed in Table 2 and Supplementary Table S2 were the most suitable. The search for polymorphic regions in nucleotide sequences of adaptively significant genes with corresponding GO terms and associated with drought resistance in *P. abies* SNP data identified 71 sequences, from which 25 contigs with the highest level of polymorphism containing several closely located SNPs were selected (Supplementary Table S3). Since the nucleotide sequences presented in the original data were too short (from 116 to 210 nucleotides) for the development of effective primers, a search for homologous sequences in the NCBI WGS database (whole-genome shotgun contigs) among those presented for *P. abies* was performed for each of the 25 sequences using BLAST+, and homologous contigs of sufficient length (from 694 to 18,086 nucleotides) were found; they were selected for the development of 25 pairs of primers (Table 2; Supplementary Table S3).

A search in the *Picea abies* genome was performed using virtualPCR and BLAST 2.2.26+ to check the uniqueness of the sequences amplified by the developed primers. As a result, primers with more than one homologous fragment for the amplicon were excluded. The remaining 16 pairs of primers (Table 3; Supplementary Tables S4–S6) were selected and tested to analyze the nucleotide diversity of the spruce complex *P. abies*–*P. obovate*.

**Figure 2:**
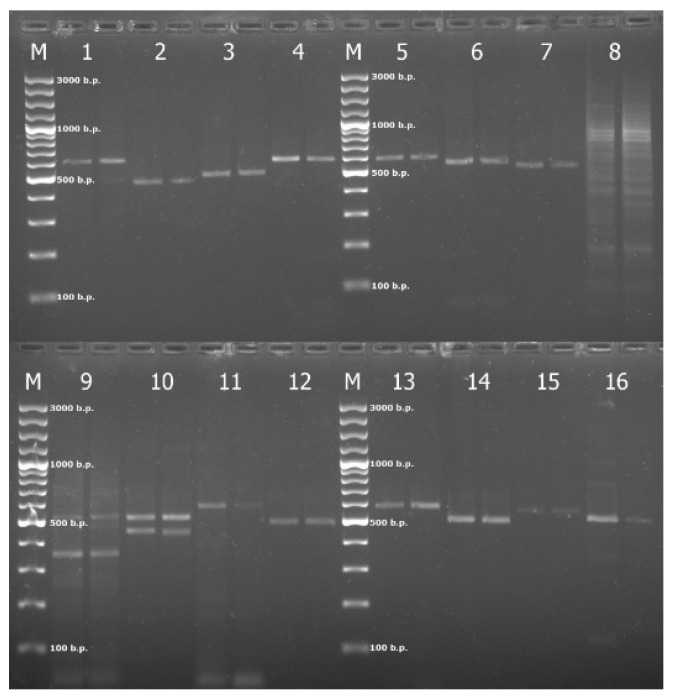
Testing the primers for the *P. abies*–*P. obovata* complex with DNA samples from the *Po_Kg* population; M—the molecular weight marker, and 1–16 are the *Pic01*–*Pic16* primers, respectively.

For experimental verification of the primers’ efficiency, test amplification was carried out with the 16 developed primer pairs. As a result, eleven primer pairs for the loci *Pic01*, *Pic02*, *Pic03*, *Pic04*, *Pic05*, *Pic06*, *Pic07*, *Pic11*, *Pic12*, *Pic13*, and *Pic14* resulted in positive amplification of single DNA fragments of the expected size (Figure 2).

The remaining loci were not amplified, which led to the amplification of non-specific fragments or did not reveal amplicons of the desired size. For this reason, they were excluded from further study. The main reason for the absence or non-specific amplification may be the incomplete affinity of the primers with the DNA of the species under study. In addition, an excessive or insufficient primer concentration, non-optimal annealing temperature, magnesium ion concentration, or the amount of DNA matrix can provoke the synthesis of non-specific fragments [33].

Therefore, to obtain high-quality target amplicons, the PCR conditions were optimized. For that purpose, the proportions and concentrations of the components in the PCR mixture and the annealing temperature in several repeated PCRs for each tested locus were varied. As a result, successful PCR amplification of a single fragment via electrophoresis was shown by primers *Pic01*, *Pic02*, *Pic04*, *Pic06*, *Pic13*, and *Pic14* (Figure 2), which were selected for further analysis.

### 3.2. SNP Position Detection and Their Analysis

**Figure 3:**
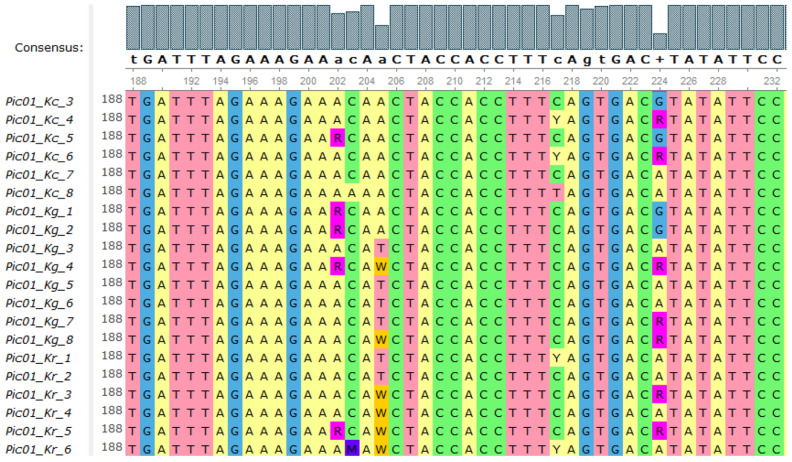
SNPs for the *Pic01* locus at the positions 202, 203, 205, 217, and 224.

As a result of sequencing, 330 nucleotide sequences of six loci were determined. The total length of the sequences was 147,822 nucleotides. The sequence alignment length varied from 390 nucleotides for the *Pic04* primer to 535 nucleotides for the *Pic01* primer. The total length of the analyzed sequences for the six loci for each sample was 2673 nucleotides. A search for sequences homologous to those obtained in the NCBI and UniProtKB databases revealed that the *Pic02*, *Pic06*, *Pic13*, and *Pic14* loci are partially similar to the known genes of other species; no annotated sequences were found for the *Pic01* and *Pic04* loci (Supplementary Table S4). The multiple alignments of the obtained nucleotide sequences resulted in a total of 81 SNPs (with a rare allele share above 1%); according to the results of multiple alignment, the *Pic06* locus is the most conservative, since in its sequence, 6 low-frequency (3–8%) SNPs were found, and the most significant number of polymorphic sites was identified in the *Pic01* locus: 27 SNPs with frequencies from 1.7 to 47.5% (Figure 3; Supplementary Table S7).

### 3.3. Use of Developed Primers for Detection of Nucleotide Polymorphism of Spruce Trees

In total, 110 polymorphic positions (including all SNPs and substitutions with a frequency of less than 1%) were found in the studied sequences of six spruce loci. The most conservative loci were *Pic02*, *Pic04,* and *Pic06*, and 12, 11, and 14 nucleotide substitutions were found in their sequences, respectively. The largest polymorphic sites (*S*) were found in the *Pic01* locus: 31 substitutions. The *Pic01* locus showed the largest number of haplotypes (*h_n_*) and polymorphic sites (*S*) in the *Po_Br* population, and the lowest values of these indicators were established in the *Po_Ch* population at the *Pic06* locus (Table 4).

The overall haplotype diversity (*Hd*), a measure of the uniqueness of a particular haplotype in a population, for the six loci studied varied from 0.516 (*Pic06* locus) to 0.947 (*Pic01* locus), and the indicator was 0.761 on average (Table 5). The nucleotide diversity index (*π*), defined as the average number of pairwise nucleotide differences per site between two DNA sequences, was higher at the *Pic01* locus (π = 0.011) and lower at the *Pic06* locus (*π* = 0.002), and the average for the six loci was 0.005. Watterson’s estimator or nucleotide diversity, calculated from the number of mutations (*θ_W_*), also revealed the highest values at the *Pic01* locus (*θ_W_* = 0.011) and lower values at the *Pic02* (*θ_W_* = 0.006), *Pic04* (*θ_W_* = 0.006), and *Pic06* (*θ_W_* = 0.006) loci. This indicates that *Pic06* is the most conservative of the six loci studied (Table 5). Watterson’s estimator exceeded the nucleotide diversity indices *π* in five of the six loci studied, which indicated an excess of low-frequency alleles in those loci and was consistent with the negative values of the Tajdima D-test (*D_T_*). The closest-to-zero *D_T_* value (−0.406) was found at the *Pic01* locus, so it can be assumed that the polymorphism of this locus is the most selectively neutral of all those studied. The most significant deviation from the neutral value (*D_T_* = −1.931) was found at the *Pic06* locus, which indicated a potentially high adaptability of the polymorphism of that locus, and in combination with its conservatism, it can be assumed that selection influences the variability of this gene (Table 5).

The nucleotide diversity data of the studied populations, revealed in the analysis of the six loci, showed similar values among the studied spruce populations; the lowest values of total haplotype diversity were found in the *Po_Kg* population (*Hd* = 0.696) and the highest in the *Po_Kc* population (*Hd* = 0.823). The most genetically heterogeneous are the *Po_Kr*, *Po_Pr*, *Po_Kc*, and *Po_Br* populations, and the *Po_Kv* and *Po_Kg* populations have less diversity (Table 6).

## 4. Discussion

The study showed that world science has accumulated a lot of data on nucleotide polymorphisms of conifers and, in particular, species of the genus *Picea* [34]. However, only a few genes among the possible adaptively significant genes can be identified as candidates for assessing the adaptability of spruce to climate change conditions and increasing drought resistance. Moreover, the data on the complete genome obtained by Nystedt et al. and others are poorly annotated, and the SNPs collected in their work generally do not carry exact data on their associative roles in resistance and sensitivity to environmental factors, including climatic conditions. This remains the main problem in understanding the patterns of formation and inheritance of conifers’ adaptations in a changing climate. Having studied the available spruce whole-genome data, we could identify less than three dozen (Table S3) sequences containing SNPs that satisfy those conditions with some degree of probability.

We found 110 polymorphic positions in the six studied spruce loci, including all single-nucleotide polymorphisms and substitutions with a frequency of less than 1%, which can be attributed to random point mutations, and only 81 single-nucleotide substitutions (about 74%) occurred with a frequency greater than 1% and can be used as SNP markers for the analysis of their adaptive role in the spruce genome. Further analysis of the detected SNPs showed that *Pic06* is the most conservative locus, with 6 low-frequency (3–8%) SNPs, and only 14 nucleotide polymorphisms were found in its sequence, and other estimates of the variability of that locus had the lowest values (*Hd* = 0.516; *π* = 0.002; *θ_W_* = 0.006). At the same time, the polymorphism of this locus is characterized by the greatest deviation from the neutral value (*D_T_* = −1.931) of the Tajima test (Table 5). Assessing the neutrality of an existing polymorphism, there are three possible interpretations of the results of the Tajima test: (1) *D_T_* = 0, with *π* = *θ_W_*; that is, the observed values of nucleotide diversity are equal to the expected ones, the population is developing in genetic equilibrium, and there is no expected influence of selection; (2) *D_T_* < 0, with *π* < *θ_W_*, which indicates an excess of low-frequency polymorphisms, which may be caused by negative selection or an increase in the population size after its recent reduction: the “bottleneck” effect; a connection with the adaptability of the gene is likely; and (3) *D_T_* > 0, with a *π* > *θ_W_* state, indicating an excess of intermediate-frequency polymorphisms, which may be a consequence of stabilizing selection or a recent sharp decrease in the population size [31]. These values indicate a potentially high adaptivity of the *Pic06* locus polymorphism, and together with the conservatism of this locus, it is possible to assume the influence of selection on the variability of this gene and then select the obtained SNPs as candidates for studying the adaptive role of the polymorphism of this gene; this is confirmed by similar studies [35]. The *Pic06* locus intersects with an exon of a gene homologous to the mitochondrial gene encoding acetyl ornithine aminotransferase (GenBank accession XM_058003929). This protein is involved in amino acid metabolism (UniProt accession Q9M8M7), which explains its high conservatism. Functionally, ornithine aminotransferase links the stress response and nitrogen metabolism and is potentially an important element in maintaining homeostasis and regulating amino acid interconversions in a plant cell. The *Pic01* locus was the most polymorphic, both in terms of the total number of polymorphic sites (*S* = 31), the number and frequencies of SNPs (27 SNPs with frequencies from 1.7 to 47.5%), and other indicators of nucleotide diversity (*Hd* = 0.947; π = 0.011; *θ_W_* = 0.011) (Table 5; Supplementary Table S7). The polymorphism of this locus had the value of the Tajima test (*D_T_* = −0.406) that was closest to zero; therefore, it is possible to assume the greatest selective neutrality of the *Pic01* locus polymorphism. But, at the same time, a high level of polymorphism and a large frequency component of the identified SNPs of this locus make it possible to use them to assess and analyze nucleotide diversity and the differentiation of spruces at the population level, as shown in similar studies [35]. In addition, the *Pic01* locus is located in the 3′UTR region of the gene homologous to the encoding α-glucan phosphorylase (GenBank accession XP_011069887). This enzyme catalyzes glycogen catabolism and plays a central role in maintaining cellular and organismal glucose homeostasis (UniProt accession A0A6I9SKI4), which may regulate energy metabolism under water deficiency conditions. For example, it was found that in *Larix sibirica*, growth slows down during a drought to a greater extent in trees with higher individual heterozygosity, which is probably due to the redistribution of energy and internal resources towards more efficient use of water and energy and the optimization of growth in dry years [36]. The adaptation and survival strategies of coniferous tree species during drought have a different nature, as shown in the example of Scots pine: adaptation at the population level is associated with the change of generations and correction of the genotypic composition and is designed for a long-term period of implementation; individual survival during drought is ensured by seasonal restructuring of the energy system at the organismal level. During drought, the energy supply of a tree is less dependent on the external environment. For example, it has been established that meristematic cells, while aging or under conditions of deficiency, are forced to switch to an energetically less expensive path of development of cellular systems—differentiation and narrow specialization—to maintain homeostasis and viability. In this regard, studying the polymorphism of the *Pic13* locus, which is homologous to the gene encoding leucine-rich repeat receptors like serine/threonine-protein kinase BAM1 (GenBank accession XP_057833279), also appears promising. This protein, a plasma membrane component, regulates the growth and structural organization of the meristem and cell differentiation (UniProt accession O49545).

Analyzing the obtained data on the nucleotide diversity of eight populations of the *P. abies–P. obovata* spruce complex in the Urals revealed an average level of genetic diversity (*Hd* = 0.758; *π* = 0.005; *θ_W_* = 0.005; *D_T_* = −0.476), which is common for different spruce species. For example, Larsson H. et al. [37] obtained similar data on nucleotide diversity (*Hd* = 0.857; *π* = 0.005; *θ_W_* = 0.007; *D_T_* = −0.880) studying 11 genomic loci among nine populations of *P. abies*. In a study of three spruce species, *P. wilsonii*, *P. morrisonicola,* and *P. neoveitchii,* in central and western China, data closest to ours were obtained among *P. wilsonii* populations (*π* = 0.005; *θ_W_* = 0.006; *D_T_* = −0.837), while *P. neoveitchii* and *P. morrisonicola* populations were characterized by lower genetic diversity values [38]. In another study of *P. mongolica* populations from Central Mongolia, based on the polymorphisms of three chloroplast regions [39], similar values of these parameters were found (*Hd* = 0.800; *π* = 0.001; *D_T_* = −0.077). In the latest large study of the hybridization processes of two spruce species over a large part of the range of *P. abies* and the eastern range of *P. obovata* [16], changes in the total nucleotide diversity (*π*) were analyzed throughout the entire range of the two species. Globally and in line with recent estimates [40], *π* was shown to vary between 0.005 and 0.007, with the highest values found in the northeastern range for *P. obovata* and southern Scandinavia for *P. abies*, i.e., populations located in contact zones or within a large hybrid zone exhibited higher *π* values than neighboring populations, which may indicate the influence of introgression and admixture on genetic diversity. It is important that hybrid populations tended to have more homogeneous allele frequencies from one SNP to another than the nominate species. The authors concluded that hybridization contributed to range expansion and climate change tolerance of two key boreal forest tree species. A genome-wide association study of drought tolerance by linking resilient dendrophenotypes and genetic variation in a study of 11 Norway spruce geographic cultivars in Austria found associations between 29 SNPs and drought tolerance traits, tree quality, and the effects of climate on tree growth [41]. Moreover, these associations explained 11 to 43% of the trait variability. Most of these SNPs were located within gene exons, the most important of which are expressed predominantly in cambium and xylem tissues. This study confirms the high adaptive variability of Norway spruce in Central and Southeastern Europe. It demonstrates how quantitative genetic, dendroclimatic, and genomic data can be jointly used to understand the genetic basis of tree adaptation to extreme climatic conditions. The discovered associations can then be used in genomic selection to predict drought-resistant phenotypes based on multilocus genotypes [42,43,44] and to assess the state of gene pools of forest tree species populations under changing climatic conditions [45,46,47].

## 5. Conclusions

This study allowed us to discover essential adaptively significant genes associated with drought tolerance and identify SNPs with potential adaptive variability, which can potentially be used as genetic markers in forest selection to improve stress tolerance and monitor adaptive genetic variability. In the future, obtaining more data for SNP genotyping based on whole-genome sequencing is necessary. The presented research can serve as a basis for effectively detecting adaptive genetic changes in forest populations. Knowledge of their adaptive potential is required to predict the consequences of global climate change and develop conservation and forest restoration programs to mitigate its potential negative impact.

## Figures and Tables

**Table 1:** Primers were selected to analyze the polymorphisms of adaptively significant genes of the spruce complex *P. abies*–*P. obovate*.

Locus	Sequence (5′–3′) Forward/Reverse Primer	Ta (°C)	PCR Band Size (bp)
*Pic01*	GCTCGTGTGAGAAACCAGGA/TGGGAAGAGGATGCAGCATG	60	598
*Pic02*	TCGGGTCCTATTCCTGCTCA/GGAAGACTCAGCAAGCCCTT	60	514
*Pic04*	ATGCTGTGGTCTCTGCACAA/GCACGCCAGAATTGATTCCC	60	585
*Pic06*	GGGCTCCCATTGTTCTTCCA/GCTTTTGCAACTGGGAAGCA	60	531
*Pic13*	CTCGCTGCTTTCTCGAATGC/TCCGAAGCTGTATACGTCGC	60	584
*Pic14*	CCCTACCCACAGTTGAGCAG/CACTTCGATCGGATGCTCGA	60	511

**Table 2:** Contig identifiers and their corresponding GO terms were selected for primer design.

GenBank Accession	Length (bp)	Gene Ontology ID	Gene Ontology Description (GO)
CBVK0101281798.1	11,571	GO:0009414	Response to water deprivation
CBVK0101480371.1	18,086	GO:0009414	Response to water deprivation
CBVK0102174952.1	7308	GO:0009414	Response to water deprivation
CBVK0102115778.1	14,665	GO:0009414	Response to water deprivation
CBVK0102359228.1	11,630	GO:0009414	Response to water deprivation
CBVK0102081567.1	2574	GO:0009414	Response to water deprivation
CBVK0103088770.1	4831	GO:0009414	Response to water deprivation
CBVK0101156587.1	3896	GO:0009414	Response to water deprivation
CBVK0100502403.1	10,672	GO:0009414	Response to water deprivation
CBVK0101505806.1	13,982	GO:0009414	Response to water deprivation
CBVK0101210595.1	10,062	GO:0009414	Response to water deprivation
CBVK0100421303.1	3906	GO:0042631	Cellular response to water deprivation
CBVK0102037105.1	4807	GO:0009414	Response to water deprivation
CBVK0101157077.1	5795	GO:0009819	Drought recovery
CBVK0100702127.1	8366	GO:0009414	Response to water deprivation
CBVK0102666519.1	4156	GO:0009414	Response to water deprivation
CBVK0103146733.1	694	GO:0009414	Response to water deprivation
CBVK0101258025.1	6333	GO:0009414	Response to water deprivation
CBVK0101506168.1	6574	GO:0009414	Response to water deprivation
CBVK0100670468.1	16,109	GO:0009414	Response to water deprivation
CBVK0100670468.1	16,109	GO:0009414	Response to water deprivation
CBVK0100280442.1	14,474	GO:0009414	Response to water deprivation
CBVK0101156767.1	1871	GO:0009414	Response to water deprivation
CBVK0100603050.1	17,104	GO:0042631	Cellular response to water deprivation
CBVK0103731373.1	6720	GO:0042631	Cellular response to water deprivation

**Table 3:** The primers were selected for test amplification in the spruce complex *P. abies*–*P. obovate genome*.

Primer	Sequence (5′–3′) Forward/Reverse Primer	Tm (°C)	PCR Band Size (bp)
*Pic01*	GCTCGTGTGAGAAACCAGGA/TGGGAAGAGGATGCAGCATG	60	598
*Pic02*	TCGGGTCCTATTCCTGCTCA/GGAAGACTCAGCAAGCCCTT	60	514
*Pic03*	TATTCCCGACACTGATGCCG/AGACAACTGCATCCACGGAG	60	505
*Pic04*	ATGCTGTGGTCTCTGCACAA/GCACGCCAGAATTGATTCCC	60	585
*Pic05*	GCCATACAAATGACGACCGC/TTTCTGCTACAGTGGCCTCG	60	551
*Pic06*	GGGCTCCCATTGTTCTTCCA/GCTTTTGCAACTGGGAAGCA	60	531
*Pic07*	GGTGGTGTTGTGGTTGATGC/AGGTGGGAGGTGATGCAATG	60	508
*Pic08*	CAGAGGTCAAACCACTGCCA/CGCAAGTGTTGAGGAGGAGT	60	521
*Pic09*	CTGCAGTGGAAGGGCTTGTA/ACCAGAAATGGCAAGGCAGA	60	508
*Pic10*	TTCTCCTAAAGCCGCTTCCG/TGAGTCAATGGCATGCCGAT	60	519
*Pic11*	CCTTGGCAAGAGGTGGAGAG/AGACCCTCCATATGTGCCCT	60	585
*Pic12*	GCCTATCAGCATTTGCCAGC/GAGTCCGGAAAGCCTCCAAA	60	559
*Pic13*	CTCGCTGCTTTCTCGAATGC/TCCGAAGCTGTATACGTCGC	60	584
*Pic14*	CCCTACCCACAGTTGAGCAG/CACTTCGATCGGATGCTCGA	60	511
*Pic15*	TCGAATCGCCCATGATCTGG/AGCCAACGAAGAAGCGGTAA	60	560
*Pic16*	TCGGTGGATCTTGGGCTAGA/ATACGGTTAAGGGGAGGGCT	60	523

**Table 4:** Several haplotypes and polymorphic sites were identified in the six locus sequences of the studied populations.

Locus *		*Po_Ch*	*Po_Kr*	*Po_Gn*	*Po_Kc*	*Po_Br*	*Po_Kv*	*Po_Kg*	*Po_Pr*	Bceгo
*Pic01*	*h_n_*	11	10	12	14	14	9	9	10	62
	*S*	19	20	17	19	20	14	15	12	31
*Pic02*	*h_n_*	7	5	5	6	6	6	6	4	14
	*S*	7	4	5	5	5	6	5	3	12
*Pic04*	*h_n_*	8	5	3	6	5	5	4	6	11
	*S*	9	6	3	6	6	4	6	8	11
*Pic06*	*h_n_*	2	5	3	3	4	4	4	4	15
	*S*	1	5	3	2	5	3	7	3	14
*Pic13*	*h_n_*	7	7	9	10	6	5	5	8	25
	*S*	6	10	10	8	7	5	7	10	23
*Pic14*	*h_n_*	7	5	11	7	6	5	3	4	24
	*S*	9	7	13	7	6	3	3	5	19

* *h_n_*—number of haplotypes, *S*—number of polymorphic sites per locus, *Po_Ch*—Cherdynsky district, *Po_Kr*—Krasnovishersky district, *Po_Gn*—Gainsky district, *Po_Kc*—Kochevsky district, *Po_Br*—Usolsky district, *Po_Kv*—Kudymkarsky district, *Po_Kg*—Karagaysky district, and *Po_Pr*—Permsky district.

**Table 5:** Total haplotype and nucleotide diversity and neutrality test statistics for the six loci studied.

Locus	Haplotype Diversity *(Hd)* *	Nucleotide Diversity (*π*)	Watterson Estimator (*θ_W_)*	Tajima D-Test Coefficient (*D_T_*)
*Pic01*	0.947 (0.013)	0.011 (0.000)	0.011 (0.002)	−0.406
*Pic02*	0.701 (0.038)	0.002 (0.000)	0.006 (0.002)	−1.545
*Pic04*	0.785 (0.029)	0.005 (0.000)	0.006 (0.002)	−0.502
*Pic06*	0.516 (0.060)	0.002 (0.000)	0.006 (0.002)	−1.931
*Pic13*	0.843 (0.021)	0.005 (0.000)	0.009 (0.002)	−1.417
*Pic14*	0.772 (0.035)	0.004 (0.000)	0.008 (0.002)	−1.339
*All*	0.761 (0.145)	0.005 (0.003)	0.008 (0.002)	−1.190

* *Hd*—total *π*.

**Table 6:** Total haplotype and nucleotide diversity of the studied spruce populations.

Population	Haplotype Diversity (*Hd*)	Nucleotide Diversity (*π*)	Watterson Estimator (*θ_W_*)	Tajima D-Test Coefficient (*D_T_*)
*Po_Ch*	0.759	0.005	0.006	−0.486
*Po_Kr*	0.775	0.005	0.006	−0.434
*Po_Gn*	0.727	0.005	0.006	−0.692
*Po_Kc*	0.823	0.005	0.005	−0.382
*Po_Br*	0.770	0.005	0.006	−0.549
*Po_Kv*	0.731	0.004	0.004	−0.354
*Po_Kg*	0.696	0.004	0.005	−0.443
*Po_Pr*	0.773	0.004	0.005	−0.469
All	0.758 (0.038)	0.005 (0.000)	0.005 (0.001)	−0.476 (0.106)

## Data Availability

Data presented in this article are available on request from the corresponding author.

## Supplementary Materials

The following supporting information can be downloaded at https://www.mdpi.com/article/10.3390/genes15111440/s1, Table S1: Coordinates and geographic distance of the studied populations; Table S2: Drought tolerance-related Gene Ontology terms; Table S3: Features of the studied contigs; Table S4: Features of the loci studied; Table S5: Gene Ontology terms of the loci studied; Table S6: Nucleotide sequences of the loci studied; Table S7: SNP frequency.
